# Oral Health Behaviors in Very Young Children in Low-Income Urban Areas in Chicago, Illinois, 2018–2019

**DOI:** 10.5888/pcd17.200213

**Published:** 2020-12-03

**Authors:** Molly Martin, Oksana Pugach, David Avenetti, Helen Lee, Shojanny Salazar, Genesis Rosales, Nattanit Songthangtham

**Affiliations:** 1 College of Medicine, Department of Pediatrics, University of Illinois at Chicago, Chicago, Illinois; 2 Institute for Health Research and Policy, University of Illinois at Chicago, Chicago, Illinois; 3 College of Dentistry, Department of Pediatrics, University of Illinois at Chicago, Chicago, Illinois; 4 College of Medicine, Department of Anesthesiology, University of Illinois at Chicago, Chicago, Illinois

**Keywords:** oral health, dental caries, child, healthcare disparities, minority health

## Abstract

**Introduction:**

Because most data on oral health do not include infants and toddlers, we aimed to describe the oral health behaviors of low-income children younger than 3 years and determine factors associated with child tooth brushing.

**Methods:**

We obtained data from the Coordinated Oral Health Promotion Chicago study, which included 420 families with children aged 6 to 36 months and their caregivers in Cook County, Illinois. We assessed child frequency of brushing from caregiver reports and objectively determined child dental plaque scores. Significant factors associated with tooth brushing frequency and dental plaque score were identified using the Least Absolute Shrinkage and Selection Operator variable selection.

**Results:**

Mean child age was 21.5 (SD, 6.9) months, and only 45% of caregivers brushed their children’s teeth twice per day or more. The mean plaque score was 1.9 (SD, 0.6), indicating high levels of plaque. Child brushing frequency was higher when children were older; used the correct toothpaste amount; brushed for a longer duration; and when caregivers brushed their own teeth more frequently, had more help with the overall care of the child’s teeth, and had family to help. Child brushing frequency was lower for caregivers with more interference from activities of daily life. Children whose caregivers had more adult help with child brushing had better plaque scores; worse plaque scores were seen in children with higher sugary beverage and food consumption and lower household incomes.

**Conclusion:**

The tooth brushing behaviors of young children are strongly associated with those of their parents and with the level of family support for brushing. Interventions to improve brushing in young children should focus on the entire family.

**Summary**

**What is already known on this topic?**

Health disparities are well documented in the prevalence of and morbidity associated with dental caries, the most common chronic disease of childhood.

**What is added by this report?**

Most data on oral health risk and behaviors do not include infants and toddlers. We describe the oral health behaviors of children younger than 3 years and identify areas for intervention.

**What are the implications for public health practice?**

Behaviors established during early childhood set the trajectory for a lifetime. This analysis shows the importance of the family unit and social support in efforts to improve oral health outcomes for high-risk children.

## Introduction

Dental caries is the most common chronic disease of childhood, affecting over half of US children aged 6 to 8 years (1). Although treatment of caries leads to significant direct health care costs, the true costs extend beyond the health care setting. Caries is associated with impaired cognitive development, increased school absenteeism, worse school performance, increased missed work for parents, and worse quality of life (2–4). Oral health disparities are well documented, and low-income minority children experience the highest prevalence and illness from caries (5–8).

Caries risk is influenced by many factors over the life course beginning during the prenatal period (9). Most data on oral health risk and behaviors do not include infants and toddlers, even though these formative years determine the trajectory for children’s oral health (10). The largest health survey in the United States, the National Health and Nutritional Examination Survey (NHANES), captures oral health behaviors data only for children aged 3 years or older. Also lacking is a complete understanding of oral health risk factors in very young children and a reliable model to predict future caries in children. Although many of these risk factors are well documented (eg, low fluoride exposure, limited access to dental care, overconsumption of sugar-sweetened beverages) (11,12), the frequency of exposure to these risk factors in children younger than 3 years is unknown. This lack of data makes it challenging to prospectively identify children who will develop caries and become high users of tertiary oral health services such as emergency departments, urgent care clinics, and operating rooms for oral care.

In addition to limited information on oral health risk factors for children younger than 3, information on the frequency and facilitators of protective oral health behaviors in this age group is also lacking. Major health promotion efforts have been implemented to educate primary care providers and families about these protective behaviors (13,14). One of the primary recommendations is twice-daily tooth brushing with fluoridated toothpaste, shown to be a low-cost clinically effective means of reducing caries for dentate children (15). Chronic conditions such as childhood obesity and diabetes have resulted in an increased awareness of the need to reduce sugar-sweetened beverages and high-sugar foods. There is a growing emphasis on the age 1 dental visit and increased coverage for private and publicly funded dental programs (16–18). Although access to dental care theoretically has improved with expanded programs and Medicaid coverage, many barriers to accessing care persist because dental coverage does not equate to use of dental care (7). Whether increased awareness of brushing and dietary recommendations translates to more adoption of these behaviors in young children is also unknown.

To effectively implement preventive interventions to establish healthy oral care behaviors, we must first characterize the baseline oral health behaviors of young children and identify factors associated with these behaviors. The Coordinated Oral Health Promotion (CO-OP) Chicago study included 420 children aged 6 to 36 months and their primary caregivers. In this analysis we describe the children’s oral health behaviors and determine factors associated with child tooth brushing, captured as caregiver-reported brushing frequency, and observed dental plaque.

## Methods

Data were obtained from the baseline sample (N = 420 child/caregiver dyads) of the CO-OP Chicago study with the National Institute of Dental and Craniofacial Research’s Oral Health Disparities Consortium (19). To qualify, families needed to have a child aged 36 months or younger with at least 2 fully erupted central maxillary incisors. Children also had to receive medical care or services at one of the partnering Special Supplemental Nutrition Program for Women, Infants, and Children (WIC) centers or pediatric medical clinics serving low-income communities in Cook County, Illinois. Families were excluded if their primary language was not English or Spanish, the child did not live with the primary caregiver 5 days per week or more, or the child had medical conditions that interfered with routine tooth brushing. Participants were recruited by research assistants (RAs) in the 20 partnering clinics and WIC centers from January 2018 through February 2019. Families that met inclusion criteria were scheduled for an enrollment visit where the baseline data collection occurred (19).

Caregivers provided written informed consent and parental permission at the start of the enrollment visit. Child assent was waived because of child age. Institutional review boards at the University of Illinois at Chicago, the University of California San Francisco, and the Chicago Department of Public Health approved the study.

Data collection was conducted mainly in homes by paired RAs using standardized methods established by the research team (19). RAs first administered a verbal questionnaire using prompt cards that asked about the child’s and caregiver’s oral health behaviors and beliefs, other health conditions, access to care, psychosocial factors, and demographics. Child and caregiver oral health quality of life was captured using the Early Childhood Oral Health Impact Scale (ECOHIS) and Oral Health Impact Profile, respectively. Caregiver quality of life (referred to as “social functioning”), depression, anxiety, and social support were measured using Patient-Reported Outcomes Measurement Information System (PROMIS) measures. Family functioning was assessed using the Confusion, Hubbub, and Order Scale (CHAOS). RAs took photographs of the child’s teeth before and after the application of plaque disclosing solution using a standardized protocol. At the end of the visit, caregivers were asked to demonstrate how the child’s teeth are typically brushed. RAs used checklists to systematically capture duration of brushing, supplies used (eg, toothbrushes, mouthwash, floss), and parent involvement. Data were entered directly into the study’s Research Electronic Data Capture (REDCap) database. Calibrated dental clinicians, including a board-certified pediatric dentist and a registered dental hygienist, later reviewed images in the research office and scored them for plaque using the Oral Hygiene Index–Maxillary Incisor Simplified (OHI-MIS) scale. The OHI-MIS scale is a modification of the Simplified Oral Hygiene Index; the adaptions allow for plaque scoring using photographs in children with an incomplete primary dentition (20). Plaque scores of less than 0.7 are considered “good,” 0.7–1.8 are “fair,” and 1.9–3.0 are “poor” (21).

### Analyses

Demographic characteristics of children and caregivers, as well as the frequency of tooth brushing and plaque scores, were reported using counts (percentages) or mean and SD for categorical variables and median and interquartile range for continuous variables. Frequency of brushing was recoded as a continuous variable, and variables with 5 or more ordinal categories were also recoded as continuous measures. Thirty-two variables were included in the analyses based on a priori–determined potential for influence on the primary outcomes. Pair-wise correlations for most covariates were low, with few correlations being in the moderate range, specifically among the PROMIS measures (ρ < 0.80). The 2 primary outcome measures, child frequency of brushing and plaque score, were correlated at −0.11, *P* = .02. Some variables had missing values. Of 420 participant records, 369 (87.9%) had complete observational data. Variables describing observed toothpaste amount, type of toothpaste, and length of observed brushing had the most missing data points (47–48 cases, 11.2%–11.4%) due mainly to parent or child refusal of the brushing demonstration. Responses were coded as “not applicable” for some variables. For example, if a child had not yet started brushing, toothpaste use would be coded as not applicable. Those responses were then recoded as no for analysis. Household income was reported as unknown by 52% of participants, which was expected for the population. Consequently, income was used as a categorical variable, with “unknown” as a category.

The selection of significant factors associated with the 2 outcomes (frequency of tooth brushing and plaque score) was performed using the Least Absolute Shrinkage and Selection Operator (LASSO) variable selection. The LASSO is a shrinkage estimator with a variable selection. The estimator shrinks regression coefficients of some of the variables to zero, hence selecting essential variables. The penalty parameter lambda determining feature selection was chosen by tenfold cross-validation to minimize predicted mean-squared error. Model averaging is a technique based on the empirical distribution of the statistics resulting from the resampling of the original population with a replacement. We used 2-step model averaging. In the first step, the model selection using LASSO shrinkage was repeated for 1,000 samples. The procedure allowed for ranking of variable importance by reporting the percentage of time that a variable was selected into the model. This first step of model averaging produced a model that contained a large number of effects. The second step of the model averaging (ie, refitting) was used to obtain a more parsimonious model by specifying the percentage of cut-point of effects retained in the final model. After a more parsimonious model was identified, a least-squares model was fit with no effect selection on 1,000 samples with replacement, which produced an empirical distribution of the regression coefficients on which the importance of the variables was based. Because standard inference does not properly consider the model selection process in LASSO, model averaging is the preferred method to interpret the standard error of the model estimates (22). The list of essential factors for both outcomes is reported using 20% and 40% frequency selection. The lower percentage was less restrictive and allowed more variables into the model. Based on the empirical distribution, the mean value of regression coefficients with a 90% confidence interval was reported as the final model results. All statistical analyses were done by using SAS 9.4 (SAS Institute).

We also tested a full linear model, allowing all 32 variables to be present, which represented the least-biased parameter estimates. All models controlled for partnering site using a set of indicator variables. The caregiver, child, and household demographic characteristics and children’s brushing behaviors are described elsewhere (19).

## Results

The mean child age was 21.5 (SD 6.9) months, and 50.7% of children were female. Almost all children had health insurance (95.5%), which was mainly Medicaid (89.3%). Most caregivers were female (96.4%) and the biological parent (96.4%); the rest were other relatives or foster parents. Parents described themselves primarily as Black race (41.9%) or Hispanic ethnicity (52.1%). More than half reported some education after high school (52.4%), 31.4% had a high school degree or GED, and 16.2% had less than a high school education. Sixty-one percent of caregivers lived with a partner or spouse. Caregivers reported their overall health as “excellent/very good” (40.5%), “good” (39.8%), or “fair/poor” (19.8%). Caregivers reported higher social functioning (mean *t*-score, 58.0 [SD, 8.1], corresponding to 0.8 SD above normative mean (mean *t*-score, 50 [SD, 10]). Caregiver anxiety and depression symptoms were slightly better than the reference population means (mean *t*-score, 46.6 [SD, 8.1] and mean *t*-score, 46.2 [SD, 6.9], respectively).

Only 25 (6.0%) caregivers had not started brushing or wiping their children’s teeth. For the rest, 45.0% brushed their children’s teeth twice per day or more, 33.8% once per day, and 15.2% brushed sometimes but not every day. The mean OHI-MIS plaque score was in the range of “poor” at 1.9 (SD 0.6); 54.9% scored 1.9 or higher. Most caregivers did all the brushing during observations without active child participation (6.2%). Most children (74.3%) had a child-sized toothbrush, and 52.3% used toothpaste with fluoride. Some children rinsed with water (36.0%) and/or spit (25.3%) after brushing. 

Caregivers reported high oral health knowledge (mean, 4.2 [SD 0.8]), and social support was comparable to reference population means (Table 1). Half of the caregivers reported that the activities of daily life never made it difficult to care for their child’s teeth, although 26.0% of caregivers never or rarely had help caring for their children’s teeth. More than half of children (59.7%) had never been to the dentist. Exclusively drinking purchased bottled water was the most common response for drinking water source (54.6%). Exposure to sugary beverages was common, with 28.8% saying their children consumed sugary beverages once per day and 37.2% consuming sugary beverages twice per day or more. Caregivers reported major oral health challenges of their own; 56.4% said their mouth and teeth were in “fair” or “poor” condition. One quarter (25.7%) brushed less than twice per day, and 43.2% had not been to a dentist in over a year. The main reasons for caregivers not getting needed dental care in the past year were related to cost and insurance coverage.

**Table 1:** Oral Health Risk Factors of Children Aged 6 to 36 Months (N = 420),^a^ Coordinated Oral Health Promotion Chicago Study, Chicago, Illinois, 2018–2019

Risk Factor	Value
**Caregiver Oral Health**	
**Condition of mouth and teeth (n = 419)**	
Very good	30 (7.2)
Good	153 (36.5)
Fair	175 (41.8)
Poor	61 (14.6)
**Frequency of brushing**	
Sometimes, but not every day	6 (1.4)
Once per day	102 (24.3)
Twice per day	254 (60.5)
More than twice per day	58 (13.8)
**Time since last dentist visit (n = 419)**	
Never have been	4 (1.0)
≤6 months	157 (37.5)
>6 months but ≤1 year	77 (18.4)
>1 year but ≤2 years	83 (19.8)
>2 years	98 (23.4)
**Main reason for last dentist visit (n = 416)**	
Went in on own	218 (52.4)
Something was wrong	138 (33.2)
Other	60 (14.4)
**Could not get dental care in the past 12 months (n = 137)**	
Could not afford	23 (16.8)
No insurance	32 (23.4)
Insurance did not cover	45 (32.8)
Pregnant	16 (11.7)
Other	21 (15.3)
**Child Risk Factors**	
**Caregiver’s/adult’s help with brushing**	
Child does not brush	25 (6.0)
Child brushes alone	11 (2.6)
Sometimes/most of the time	151 (36.0)
Always	233 (55.5)
**Length of time since child’s last dental visit (n = 419)**	
Never has been	250 (59.7)
≤6 months	139 (33.2)
>6 months but ≤2 years	30 (7.2)
**Child needed dental care but could not get, past 12 months**	31 (7.4)
**Type of drinking water (n = 416)**	
Purchased only	227 (54.6)
Tap only	73 (17.5)
Both purchased and tap	116 (27.9)
**Frequency of sugary beverage consumption**	
Rarely or never	74 (17.6)
Once per week, not daily	69 (16.4)
Once per day	121 (28.8)
Twice per day	83 (19.8)
Three times per day or more	73 (17.4)
**Child 15 months or older and still drinks from bottle (n = 341)**	137 (40.2)
**Caregiver Knowledge, Support, and Barriers**	
**Caregiver knowledge, mean (SD)^b^**	4.2 (0.8)
**Family/partner help care for child’s teeth**	
All the time	144 (34.3)
Most of the time	83 (19.8)
Some of the time	84 (20.0)
Rarely	42 (10.0)
Never	67 (16.0)
**Social support, *t*-score, mean (SD) (n = 419)**	
Emotional	55.9 (8.9)
Instrumental	54.8 (9.3)
Informational	57.7 (9.8)
**Activities of daily life make it difficult to care for child’s teeth**	
All the time	7 (1.7)
Most of the time	29 (6.9)
Some of the time	84 (20.0)
Rarely	88 (21.0)
Never	212 (50.5)

a Values are no. (%), unless otherwise indicated; N = 420 unless otherwise indicated.

b The Oral Health Knowledge Scale was developed by the Knowledge and Behavior Workgroup of the Early Childhood Caries Collaborating Centers (31).

Tables 2 and 3 present the results of multiple linear models without selection (full models) and the variables selected using LASSO regularization from the 32 potential associated factors, controlling for partner sites. The overlap in variables identified as important between the full models and LASSO regularization indicates low confounding and multicollinearity between variables. The LASSO 20% frequency selection shows the less restricted model, which allowed more variables to remain. The important factors for frequency of child tooth brushing identified by the more restrictive LASSO regularization with a 40% threshold to be retained in the final model included 8 variables, 7 of which met significance at the 10% level. Child brushing frequency (Table 2) was higher when children were older (mean β = 0.014; 90% CI, 0.006 to 0.022); used the correct toothpaste amount (mean β = 0.115; 90% CI, 0.004 to 0.221); brushed for a longer duration (mean β = 0.001; 90% CI, 0.000 to 0.002); and when caregivers brushed their own teeth more frequently (mean β = 0.397; 90% CI, 0.299 to 0.490), had more help with the overall care of the child’s teeth and child brushing (mean β = 0.058; 90% CI, 0.021 to 0.096), and had family or a partner to help care for the child’s teeth (mean β = 0.292; 90% CI, 0.229 to 0.354). Child brushing frequency was lower for caregivers with more brushing interference from activities of daily life (mean β = −0.105; 10% CI, −0.161 to −0.048).

**Table 2:** Factors Associated with Frequency of Child Tooth Brushing Among Children Aged 6 to 36 Months (N = 420), Coordinated Oral Health Promotion Chicago Study, Chicago, Illinois, 2018–2019^a^

Variable	Full Model			LASSO 20% Frequency Selection			LASSO 40% Frequency Selection		
	β	SD	10% CI	Mean β	SD	90% CI	Mean β	SD	90% CI
Intercept	0.065	0.751	–1.170 to 1.301	–0.545^b^	0.256	–0.961 to –0.119	–0.613^b^	0.139	–0.835 to –0.381
Activities of daily life make it difficult to care for child’s teeth	–0.127^b^	0.033	–0.182 to –0.072	–0.095^b^	0.034	–0.149 to –0.039	–0.105^b^	0.034	–0.161 to –0.048
Caregiver age in years	0.002	0.006	–0.007 to 0.012	0.002	0.005	–0.006 to 0.011	—	—	—
Caregiver/adults help with brushing	0.281^b^	0.041	0.214 to 0.349	0.275^b^	0.040	0.207 to 0.340	0.292^b^	0.037	0.229 to 0.354
Caregiver frequency of brushing	0.378^b^	0.055	0.287 to 0.468	0.387^b^	0.056	0.292 to 0.477	0.397^b^	0.058	0.299 to 0.490
Child age in months	0.015^b^	0.006	0.006 to 0.025	0.014^b^	0.006	0.004 to 0.023	0.014^b^	0.005	0.006 to 0.022
Correct toothpaste amount	0.097	0.071	–0.020 to 0.214	0.093	0.073	–0.022 to 0.215	0.115^b^	0.068	0.004 to 0.221
Family/partner help care for child’s teeth	0.078^b^	0.025	0.037 to 0.118	0.058^b^	0.023	0.018 to 0.095	0.058^b^	0.023	0.021 to 0.096
Fluoride toothpaste used	0.005	0.071	–0.113 to 0.122	0.021	0.068	–0.090 to 0.131	—	—	—
Frequency of sweet or sugary foods	–0.004	0.005	–0.012 to 0.005	–0.005	0.005	–0.012 to 0.003	—	—	—
Household chaos	–0.088	0.064	–0.193 to 0.017	–0.037	0.062	–0.144 to 0.064	—	—	—
**Household income in last year, $**									
<30,000	0.091	0.081	–0.042 to 0.223	0.107	0.080	–0.024 to 0.240	0.100	0.078	–0.029 to 0.236
30,000–60,000	–0.056	0.093	–0.210 to 0.097	—	—	—	—	—	—
>60,000	–0.142	0.144	–0.379 to 0.095	—	—	—	—	—	—
Unknown/refused	1 [Reference]			—	—	—	—	—	—
Length of time since child’s last dental visit	0.065	0.056	–0.028 to 0.158	0.046	0.055	–0.047 to 0.138	—	—	—
Observed brushing time in seconds	0.001^b^	0.001	0.000 to 0.002	0.001^b^	0.001	0.000 to 0.002	0.001^b^	0.001	0.000 to 0.002

Abbreviations: —, not applicable; LASSO, Least Absolute Shrinkage and Selection Operator.

a Models include 32 variables; only significant variables are reported in the table. All models also control for a partner site. The full model uses categorical variables as a single construct, whereas LASSO treats the set of indicator variables from the same categorical variables as independent variables. The coefficients for the household income variable represent differences from the reference category in the full model, but in the LASSO models the coefficients represent differences from all categories not selected into the model.

b Significant at *P* < .10.

**Table 3:** Factors Associated with Higher Child Plaque Score Among Children Aged 6 to 36 Months (N = 420), Coordinated Oral Health Promotion Chicago Study, Chicago, Illinois, 2018–2019^a^

Factor	Full Model			LASSO 20% Frequency Selection			LASSO 40% Frequency Selection		
	β	SD	10% CI	Mean β	SD	90% CI	Mean β	SD	90% CI
**Intercept**	2.000^b^	0.628	0.684 to 2.749	1.939^b^	0.204	1.593 to 2.276	2.010^b^	0.107	1.833 to 2.181
**Caregiver/adults help with brushing**	–0.101^b^	0.040	–0.166 to –0.037	–0.102^b^	0.040	–0.167 to –0.034	–0.092^b^	0.040	–0.156 to –0.028
**Caregiver age in years**	–0.005	0.005	–0.014 to 0.004	–0.004	0.005	–0.012 to 0.004	—	—	—
**Caregiver relationship status**									
Single	0.247	0.145	0.009 to 0.484	0.114	0.071	–0.007 to 0.225	—	—	—
Living with partner/spouse	0.122	0.138	–0.104 to 0.348	—	—	—			—
Separated/divorced	1 [Reference]			—	—	—	—	—	—
**Child race/ethnicity**									
Black	0.016	0.149	–0.229 to 0.261	—	—	—	—	—	—
Hispanic	0.195	0.151	–0.053 to 0.443	0.127^b^	0.073	0.008 to 0.252			—
Other	0.391	0.249	–0.020 to 0.801	0.347^b^	0.181	0.040 to 0.628			—
White	1 [Reference]			—	—	—	—	—	—
**Observed brushing time in seconds**	–0.001^b^	0.001	–0.002 to –0.000	–0.001^b^	0.001	–0.002 to –0.000	–0.001	0.001	–0.002 to 0.000
**Fluoride toothpaste used**	0.098	0.069	–0.015 to 0.211	0.109^b^	0.065	0.002 to 0.218	—	—	—
**Frequency of sugary beverage consumption**	0.014^b^	0.005	0.006 to 0.023	0.014^b^	0.005	0.005 to 0.022	0.014^b^	0.005	0.006 to 0.022
**Frequency of sweet/sugary foods**	0.008	0.005	–0.000 to 0.016	0.007	0.005	–0.001 to 0.015	0.009^b^	0.005	0.001 to 0.017
**Household income in last year, $**									
<30,000	0.173^b^	0.076	0.047 to 0.300	0.175^b^	0.074	0.049 to 0.292	0.153^b^	0.071	0.036 to 0.268
30,000–60,000	–0.011	0.090	–0.157 to 0.135	—	—	—	** —**	** —**	** —**
>60,000	0.022	0.136	–0.246 to 0.202	—	—	—	** —**	** —**	** —**
Unknown/refused	1 [Reference]			—	—	—	** —**	** —**	** —**
**Total ECOHIS Score**	0.009	0.007	–0.004 to 0.021	0.005	0.007	–0.006 to 0.017	—	—	—

Abbreviations: —, not applicable; ECOHIS, Early Childhood Oral Health Impact Scale; LASSO, Least Absolute Shrinkage and Selection Operator.

a Models include 32 factors; only significant variables are reported in the table. All models also control for a partner site. The full model uses categorical variables as a single construct, whereas LASSO treats the set of indicator variables from the same categorical variables as independent variables. In this model, for caregiver race/ethnicity, caregiver relationship status, and household income, the coefficients in the full model represent differences from the reference category but in the LASSO models, the coefficients represent differences from all categories not selected into the model.

b Significant at *P* < .10.

With regard to plaque score, children whose caregivers had more help from other adults with brushing their child’s teeth had better plaque scores (mean β = −0.092; 90% CI, −0.156 to −0.028) (Table 3). Higher plaque scores were seen in children with higher sugary beverage consumption (mean β = 0.014; 10% CI, 0.006 to 0.022), higher sweet or sugary food consumption (mean β = 0.009; 90% CI, 0.001 to 0.017), and lower household incomes (mean β = 0.153; 90% CI, 0.036 to 0.268).

## Discussion

We identified multiple factors associated with tooth brushing behaviors and dental plaque in low-income children aged 36 months or younger, and these findings are relevant because dental caries begins early. The consistency in selecting the same set of factors between the full and LASSO regularization models highlights the robustness of the selection procedure in identifying meaningful factors associated with the frequency of tooth brushing and plaque score for this population. 

Data from 2011–2016 reported a caries prevalence of 23% in US children aged 2 to 5 years, and this prevalence doubled by elementary school (1). In Illinois, overall caries and untreated caries prevalence have repeatedly surpassed national rates and disproportionately impact low-income, non-Hispanic Black, and Hispanic children (23). The participants in our analyses represent this high-risk demographic category. Identification of early risk and protective factors is essential to reduce oral health disparities and prevent or slow caries development in children.

The most influential factors associated with child brushing frequency in our analyses were the caregiver’s own brushing frequency and caregivers having assistance with brushing from others. These associations are consistent with findings from other studies (24–26). Caregivers that brush their own teeth are more likely to brush their children’s teeth as well (26). This association may be driven by caregiver oral health literacy, an overall value on oral hygiene within the family, established household routines, or by the fundamental principle that children learn from imitating adults (27). Having additional caregivers assist with child tooth brushing was associated with both higher brushing frequency and lower plaque scores. This points to the critical need for more family support for child brushing at this young age, mainly because children do not have the knowledge or manual dexterity to brush their teeth independently until they are much older. Caregivers have a fixed amount of time to complete necessary tasks, such as those conducted as part of morning or evening routines. When additional caregivers are available to assist with these tasks, children are more likely to receive assistance or supervision with an oral health regimen. Our findings emphasize the importance of the family unit or household, as everyone plays an important role in encouraging and directly supervising a child’s tooth brushing.

The frequency of consumption of sugary foods and beverages was associated with worse plaque scores. This finding may be because parents that give more sugary beverages may demonstrate other unhealthy behaviors such as brushing less frequently or effectively. Households that rely on calorie-dense, readily available foods may do so out of necessity and not have the capacity or support to implement regular brushing routines. This finding is concerning because the frequency of exposure to sugar-sweetened foods and beverages is a significant risk factor for dental caries via acidogenic bacteria in plaque (28).

Finally, children in the lowest income category had the highest levels of plaque. Although most of our sample was low-income, worse outcomes in the lowest income level are not surprising. Low-income caregivers have repeatedly reported significant barriers to accessing dental care for their children (29), and these results are compounded by the lack of providers that accept Medicaid, as well as limited case management resources. The overall rate of providers enrolled with Medicaid in the Chicago area is high compared with the rest of the state, mirroring the geographic density of Illinois’s population. Unfortunately, enrollment as a Medicaid provider does not mean these dentists serve a significant number of patients on Medicaid. The reality is that many of these providers take only a small number of Medicaid patients and may not perform restorative procedures.

What was surprising were the many factors not associated with brushing behaviors, including access to dental care, caregiver quality of life, social functioning, and caregiver oral health knowledge. Research indicates that children’s dental care usage behaviors were associated with their caregivers’ behaviors in these areas; children were more likely to have used dental care within the past year when their parents also used dental services (23,25). A possible explanation for the lack of association between dental care use and child brushing behaviors is that most of our sample had the same insurance, limiting variability. Research shows that the overall physical and psychological health and functioning of caregivers influences how they care for the health of their children; poor health, adversity, and inequality accumulate over the life course and across generations (30). Our study did not show differences in behaviors associated with caregiver quality of life and mental health, which may have been because of a lack of variability in the sample, instrument limitations, or perhaps not-yet-identified resilience factors. Finally, uncooperative child behavior is common in toddlers and poses a barrier to tooth brushing, even when caregiver knowledge and intent are good.

Our study has limitations. Because the data were cross-sectional, causation and potential directionality of effects cannot be established. We also did not measure all modifiable factors that influence oral health behaviors. The sample was limited to 1 densely populated urban county in the Midwest, and families had similar economic and races/ethnicities, which limits generalizability. Tooth brushing frequency was caregiver-reported because of the challenges of objectively measuring this behavior in young children, raising the potential for social desirability bias and data inaccuracy. However, we compared our self-reported data to data from other studies, including NHANES, and our results were similar (19). We also added a second measure of brushing — plaque score — to objectively capture the adequacy of brushing behaviors.

Our results indicate the necessity of interventions that target adult assistance with child brushing and reduction of sugary beverages and snack consumption among very young children. Similar to results in older children, our results demonstrate that brushing behaviors of young children are strongly associated with those of their parents and the level of family support for brushing. Interventions to improve brushing in young children should focus on the entire family, encouraging healthy oral health behaviors for parents as well as children. Clinicians and educators should also consider asking about family routines and supports parents have for brushing their children’s teeth and offering appropriate interventions when problems are identified. Because low-income urban children are at high risk for developing caries beginning at a very early age, research is needed to determine whether these risk factors are also associated with caries development over time. We should also continue to develop and test interventions that will translate into improved oral health behaviors and outcomes for children.
