# Erratum, Vol. 17, December 3 Release

**DOI:** 10.5888/pcd23.200213e

**Published:** 2026-05-21

In the article “Oral Health Behaviors in Very Young Children in Low-Income Urban Areas in Chicago, Illinois, 2018–2019,” one of the measures used (PROMIS Participation in Social Activities) was incorrectly scored. The affected data in the tables have been updated, and these changes do not change the primary findings of the study. The analysis was to identify factors associated with oral health behaviors, and this variable was not associated, either before or after the error was fixed.

The correction was made to our website on May 8, 2026, and appears online at https://www.cdc.gov/pcd/issues/2020/20_0213.htm. We regret any confusion or inconvenience this error may have caused.

